# Perceptions of Racially and Ethnically Diverse Women at High Risk of Breast Cancer Regarding the Use of a Web-Based Decision Aid for Chemoprevention: Qualitative Study Nested Within a Randomized Controlled Trial

**DOI:** 10.2196/23839

**Published:** 2021-06-08

**Authors:** Tarsha Jones, Ashlee Guzman, Thomas Silverman, Katherine Freeman, Rita Kukafka, Katherine Crew

**Affiliations:** 1 Florida Atlantic University Boca Raton, FL United States; 2 Columbia University New York, NY United States

**Keywords:** breast cancer, chemoprevention, qualitative, decision support, cancer, estrogen receptor, web-based, cancer risk

## Abstract

**Background:**

Chemopreventive agents such as selective estrogen receptor modulators and aromatase inhibitors have proven efficacy in reducing breast cancer risk by 41% to 79% in high-risk women. Women at high risk of developing breast cancer face the complex decision of whether to take selective estrogen receptor modulators or aromatase inhibitors for breast cancer chemoprevention. *RealRisks* is a patient-centered, web-based decision aid (DA) designed to promote the understanding of breast cancer risk and to engage diverse women in planning a preference-sensitive course of decision making about taking chemoprevention.

**Objective:**

This study aims to understand the perceptions of women at high risk of developing breast cancer regarding their experience with using *RealRisks*—a DA designed to promote the uptake of breast cancer chemoprevention—and to understand their information needs.

**Methods:**

We completed enrollment to a randomized controlled trial among 300 racially and ethnically diverse women at high risk of breast cancer who were assigned to standard educational materials alone or such materials in combination with *RealRisks*. We conducted semistructured interviews with a subset of 21 high-risk women enrolled in the intervention arm of the randomized controlled trial who initially accessed the tool (on average, 1 year earlier) to understand how they interacted with the tool. All interviews were audio recorded, transcribed verbatim, and compared with digital audio recordings to ensure the accuracy of the content. We used content analysis to generate themes.

**Results:**

The mean age of the 21 participants was 58.5 (SD 10.1) years. The participants were 5% (1/21) Asian, 24% (5/21) Black or African American, and 71% (15/21) White; 10% (2/21) of participants were Hispanic or Latina. All participants reported using *RealRisks* after being granted access to the DA. In total, 4 overarching themes emerged from the qualitative analyses: the acceptability of the intervention, specifically endorsed elements of the DA, recommendations for improvements, and information needs. All women found *RealRisks* to be acceptable and considered it to be helpful (21/21, 100%). Most women (13/21, 62%) reported that *RealRisks* was easy to navigate, user-friendly, and easily accessible on the web. The majority of women (18/21, 86%) felt that *RealRisks* improved their knowledge about breast cancer risk and chemoprevention options and that *RealRisks* informed their (17/21, 81%) decision about whether or not to take chemoprevention. Some women (9/21, 43%) shared recommendations for improvements, as they wanted more tailoring based on user characteristics, felt that the DA was targeting a narrow population of Hispanic or Latina by using graphic novel–style narratives, wanted more understandable terminology, and felt that the tool placed a strong emphasis on chemoprevention drugs.

**Conclusions:**

This qualitative study demonstrated the acceptability of the *RealRisks* web-based DA among a diverse group of high-risk women, who provided some recommendations for improvement.

## Introduction

Breast cancer is the most commonly diagnosed cancer among women in the United States and the second leading cause of cancer-related deaths; therefore, prevention of the disease would significantly improve public health [1]. An estimated 268,600 new cases of invasive breast cancer occur among women each year, and 41,769 women will die from the disease [1,2]. The United States Preventive Services Task Force recommends that clinicians offer chemoprevention as a primary prevention strategy to women at high risk of breast cancer and low risk of adverse effects from these drugs [3]. High-risk criteria for breast cancer are defined as a 5-year invasive breast cancer risk of ≥1.67% or lifetime risk score of ≥20% according to the Gail risk model, which accounts for age, race and ethnicity, benign breast disease, first-degree family history of breast cancer, and reproductive factors [4,5]. Chemopreventive agents such as selective estrogen receptor modulators (tamoxifen and raloxifene) and aromatase inhibitors (exemestane and anastrozole) have been shown to reduce breast cancer risk by 41%-79% among high-risk women [6]. Despite the risk-reducing benefits, chemoprevention uptake remains low in the United States, with fewer than 5% of high-risk women deciding to take the medication [4]. Racial and ethnic minority women are less likely to seek breast cancer preventive care [7,8], which contributes to higher rates of late-stage diagnoses, poorer clinical outcomes, and health disparities [9-11].

Patient-level barriers to chemoprevention uptake include inadequate knowledge and negative attitudes, the fear of potential side effects, and inaccurate perceptions of breast cancer risk [4,12]. Previous studies have found that some women are not aware of the availability of chemoprevention drugs, and less awareness has been noted among racial and ethnic minority women [12]. Women may also be skeptical about the efficacy of chemoprevention in reducing the risk of breast cancer [12]. Studies have found that the fear of the potential side effects of tamoxifen leads to negative attitudes toward chemoprevention, including perceptions that the increased risks of endometrial cancer, pulmonary embolism, stroke, deep vein thrombosis, cataracts, hormonal symptoms, and sexual problems outweigh the potential benefits of the drugs (ie, reduced risks of breast cancer and osteoporosis) [13-16]. In addition, when women view themselves as healthy and not at high risk of developing breast cancer, they are less likely to take chemoprevention drugs [17].

Women at high risk of developing breast cancer face the complex decision of whether to take selective estrogen receptor modulators or aromatase inhibitors for breast cancer chemoprevention [5,12]. The decision is complex because (1) the efficacy of these drugs in preventing breast cancer is limited to estrogen receptor–positive tumors, (2) there is an increased risk of developing serious medical conditions with the use of these drugs, and (3) the recommendations are different for pre- and postmenopausal women [5,12]. Therefore, the best choice for chemoprevention is not always clear, making this a preference-sensitive decision that takes into account how each individual values the relative potential benefits and harms [5,12]. Several interventions have been designed to increase chemoprevention uptake [14,15,18-20]. However, these interventions have been met with limited success, ranging from 0.5% to 5.6% chemoprevention uptake [14,15,18-20]. To address patient-related barriers to chemoprevention among racially and ethnically diverse women, our research team developed a patient-centered, web-based decision aid (DA)*, RealRisks,* which is currently being tested in 3 clinical trials [4,21,22].

The purpose of this study is to understand the perceptions of women at high risk of developing breast cancer regarding their experience with using *RealRisks*—a DA designed to promote the uptake of breast cancer chemoprevention—and to understand their information needs.

## Methods

### Study Design

We conducted a qualitative study to understand high-risk women’s perceptions about *RealRisks.* This study was nested within a randomized controlled trial (RCT) that involved 300 high-risk women assigned to standard educational materials alone or in combination with *RealRisks* to determine chemoprevention uptake at 6 months (primary outcome) [4]*.*

### Intervention

**Figure 1:**
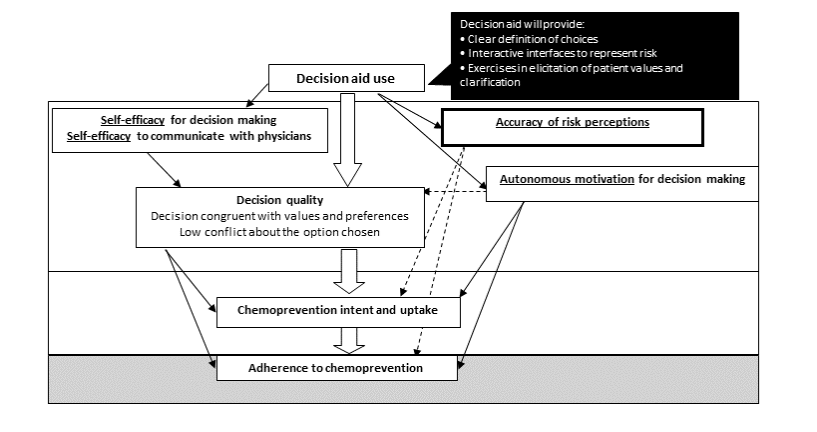
Multidisciplinary framework based on shared decision making and self determination theory.

*RealRisks* was designed to promote a woman’s understanding of her breast cancer risk and to engage women in planning a preference-sensitive course of decision making about chemoprevention. This web-based DA incorporates 2 complementary theoretical frameworks—shared decision making [23] and self-determination theory (SDT) [24]—to engage women in planning a preference-sensitive course of decision making about chemoprevention. SDT has at its core the concept of autonomous motivation [24-26] and describes autonomous choices as those that a person could fully endorse upon reflection [27-29]. This is contrasted with behaviors or choices that feel *controlled* or coerced by another person. Fully autonomous choices involve reflecting on and integrating one’s preferences and values. The key is to facilitate choice in the context of decisions concerning chemoprevention, in a manner such that the evidence presented is not experienced as coercive but as supportive of autonomous choice. As shown in Figure 1, the *RealRisks* DA is intended to promote the accuracy of breast cancer risk perceptions, autonomous motivation, self-efficacy for decision making, and chemoprevention uptake.

**Figure 2:**
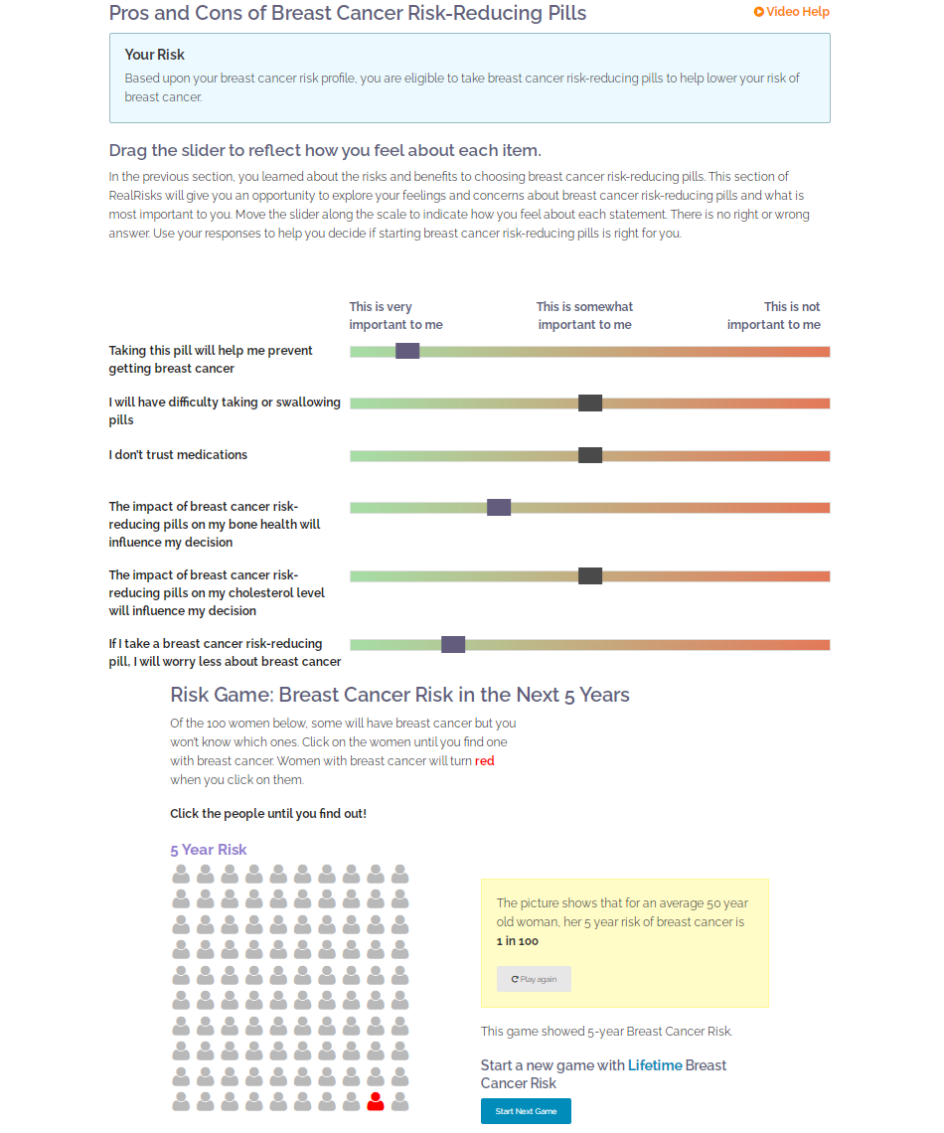
Screenshots of RealRisks.

The DA is delivered via audio files with Spanish translation and is organized into the following modules: (1) breast cancer risk (breast cancer risk factors, calculation of personal breast cancer risk, and interactive games on risk communication), (2) chemoprevention, (3) family history and genetic testing, and (4) lifestyle factors. The content in *RealRisks* has been tailored to a Hispanic or Latina group of women. Figure 2 shows the interactive components of the tool that the women complete during their engagement with the tool. The DA has been described in detail in previous publications [4,22].

### Participants and Settings

Interview participants were recruited from the intervention arm of the RCT. Participants were eligible if they (1) were English or Spanish speaking, (2) completed their 6-month survey, and (3) had access to the web-based *RealRisks* DA through the intervention arm of the RCT. Recruitment and data collection occurred between February and April 2020. Eligible participants were recruited via email and telephone interviews. The research team stopped recruitment efforts upon reaching data saturation—the point at which the collected data stopped producing novel insights [30]. The study protocol was approved by the institutional review board at Columbia University and Florida Atlantic University.

### Data Collection

Eligible participants were invited to participate in a one-on-one semistructured interview conducted over the telephone or via Zoom web conferencing technology (Zoom Video Communications). The interviews were audio recorded to ensure accuracy. A semistructured interview guide was developed with open-ended questions to explore women’s perceptions and experiences using *RealRisks.* We explored participants’ acceptability of the tool (what they liked or disliked about *RealRisks* and what new information they learned) and any unmet information needs after interacting with the tool. We also explored participants’ access to electronic devices used to access the tool and any technological issues that participants may have encountered. In addition, we asked women about their decision making regarding chemoprevention, factors impacting their decisions, and their clinical encounters with their health care provider. A total of 2 versions of the interview guides were developed: one for women who used *RealRisks* and another for women who had never accessed the DA and could not recall using *RealRisks.* The interviews were conducted by TJ and AG and lasted between 20 and 60 minutes. The interviews were transcribed verbatim by a Health Insurance Portability and Accountability Act–compliant transcription company and compared with the original digital recording to ensure the accuracy of the content. This paper reports data on the perceptions, experiences, and information needs of high-risk women who were granted access to the DA through the intervention arm of the parent trial.

### Data Analysis

Transcribed and deidentified data were analyzed using Dedoose software (SocioCultural Research Consultants). Content analysis was used to systematically describe the meaning of the qualitative data [31,32]. Data analyses were performed in several steps. First, the principal investigator (TJ) read the first 2 transcripts to gain familiarity with the data and used open coding to build a coding framework derived from the interview guide topics. Second, the analysis team, consisting of researchers trained in conducting qualitative research (TJ, AG, and TS), coded the first 2 transcripts independently using line-by-line coding and discussed code applications as a group to develop consensus. Third, the analysis team members used the Dedoose training center to evaluate interrater reliability by generating a pooled Cohen κ coefficient to assess coding precision [33]. The final pooled Cohen κ score was 0.86, which indicated a high level of coding agreement among the coding team [34]. Fourth, TJ and AG continued to code the remaining transcripts independently and used the memo feature in Dedoose to review each other’s coding and to capture thoughts and observations within and between transcripts. Finally, the analysis team abstracted and interpreted the data to generate thematic domains. To ensure trustworthiness, direct quotations were provided to connect the results to the raw data. Ellipses were used (3 periods indicating a break within a sentence) to help minimize the length of the quotations. In addition, some larger quotations were presented to keep the context of the conversation intact.

## Results

### Participants and Interview Data

A total of 21 high-risk women participated in this qualitative study (Table 1). The mean age of the participants was 58.5 (SD 10.1) years. Our sample was racially and ethnically diverse, with demographics distributed as follows: 5% (1/21) Asian, 24% (5/21) Black or African American, and 71% (15/21) White. In addition, 2 participants were Hispanic or Latina. Most (15/21, 71%) participants had a family history of breast cancer, and all women (21/21, 100%) reported using *RealRisks* after being granted access to the DA. Interview data were categorized into 4 main themes: (1) the acceptability of the intervention, (2) specifically endorsed elements of the DA, (3) recommendations for improvement, and (4) information needs. Each theme comprised several subthemes, and exemplar quotes are presented to authenticate the overarching themes and subthemes. Themes, subthemes, and exemplar quotes are also provided in Multimedia Appendix 1.

**Table 1:** Study participant characteristics (N=21).

Characteristics			Value
Age (years), mean (SD)			58.5 (10.1)
**Ethnicity, n (%)**			
	Hispanic or Latina	2 (9)	
	Non-Hispanic	19 (91)	
**Race, n (%)**			
	Asian	1 (5)	
	Black or African American	5 (24)	
	White	15 (71)	
**Highest level of education, n (%)**			
	High school or GED^a^	2 (11)	
	Associates or bachelors	9 (50)	
	Some college	2 (11)	
	Graduate degree	5 (28)	
**Primary language, n (%)**			
	English	21 (100)	
**Family history of breast cancer, n (%)**			
	Yes	15 (71)	
	No	6 (29)	
**Taking chemoprevention, n (%)**			
	Yes	20 (95)	
	No	1 (5)	
**Used the RealRisks decision aid, n (%)**			
	Yes	21 (100)	
	No	0 (0)	

^a^GED: General Education Development.

### Theme 1: The Acceptability of the Intervention

#### Subtheme 1: General Perceptions

All women (21/21, 100%) who accessed *RealRisks* shared their general perceptions that they liked using the DA and considered it to be helpful. Almost 80% (16/21, 76%) of the women who were granted access to the DA through the intervention arm of the parent trial completed the tool. Some women (9/21, 43%) shared recommendations for improvement after using the tool. Overall, women from all 4 racial and ethnic groups represented in our study viewed the tool as acceptable. One of the women stated:

I felt that it was good for me to have done it [RealRisks]. And I do feel that I got information that I would not have gotten otherwise. I mean, I felt a little bit more informed. So, I’m happy about that.

Participant #18

#### Subtheme 2: Usability of the Intervention

Most women (13/21, 62%) felt that *RealRisks* was easy to navigate. For instance, women shared the following:

It was pretty simple and easy to do, and I didn’t feel like there were a lot of issues with using the tool and taking the surveys at all...I didn’t find it to be off putting. I didn’t find it to be hard to do. I didn’t find it to suck up a lot of my time.

Participant #27

It was easy to access. The instructions were pretty good...Simple and basic and not overwhelming and I like the fact that if you didn’t know something you could just hover over a term and you could get a definition.

Participant #26

I liked the fact that it was quick. I was able to just go through it.

Participant #21

I thought it was easy to use. It answered the questions that I would have. Like it was very thorough in the questions, so I didn’t have to wonder what it meant. It wasn’t up for interpretation. I thought it was pretty clear.

Participant #25

None of the women who used the DA reported encountering any technological issues. One woman stated:

No. I was able to get in. I was able to answer all the questions. I don’t remember any glitches at all.

Participant #15

#### Subtheme 3: User-friendly

Other women felt that the DA was user-friendly, straightforward, simple to follow, and easy to understand. They shared the following:

It was pretty easy to understand. I have no medical background and it gave, I thought, very clear, information about how different drugs could help. And then I could answer the questions that came next.

Participant #23

Pretty easy. Sort of user-friendly in terms of just accessing all the steps.

Participant #18

It was simple to follow-simple to use. It didn’t take up too much time. I mean, it’s been a while. It’s been over a year, right? But I remember just being able to just quickly go on my tablet and answer the questions.

Participant #7

I thought it was easy to understand and to complete. I thought it was pretty user-friendly for someone like myself.

Participant #5

#### Subtheme 4: Easily Accessible on the Web

Most women (19/21, 90%) reported having access to technological devices such as cell phones, tablets, laptops, and desktop computers to access *RealRisks.* The exceptions were 2 Spanish-speaking participants who reported having no access to a computer at home and came to the hospital to receive in-person support to access the tool from one of our research team members. The women who had access to technology liked that the DA was web-based, which allowed them to access it on the web at their own convenience. For example, women shared the following:

It was easy because I could do it online. You know, I could like start it at work. I could finish it at home. I did not have to go back and re-answer questions. I thought it was user-friendly.

Participant #1

I could do it online, could start it at work and finish it at home.

Participant #2

It was good that it was online and it was easy to navigate.

Participant #14

I didn’t really have any trouble following through. But, you know, I guess, with everything that’s done online, you’re doing it on your own time.

Participant #18

### Theme 2: Specifically Endorsed Elements of the DA

#### Subtheme 1: Improved Knowledge

Most women (18/21, 86%) described gaining new knowledge after using *RealRisks*. A total of 11 women described learning new information about breast cancer prevention drugs. The participants described learning new information such as which chemoprevention drug was appropriate for them, for example, tamoxifen and other available drugs. Other women described learning which drug was appropriate for premenopausal and postmenopausal women. The women described the following:

New information was the information around the drugs to take. I can’t remember what they were called, but the preventive, I guess that’s what they are, drugs.

Participant #5

I knew about the existence of the drug, tamoxifen. I know it opposes estrogen. An anti-estrogen-type drug. I did not know about other drugs. So that was new information.

Participant #24

In total, 2 of these women explained that *RealRisks* reinforced some of the information that they had already heard from their health care providers, which contributed to an improvement in their overall knowledge:

Being diagnosed with the BRCA2 gene mutation, I was getting so much information at that time and doing so much research and meeting with so many different doctors because of trying to be very, very proactive about being diagnosed with the BRCA2 gene that it actually turned out to have been helpful in terms of reinforcing some ideas that I didn’t necessarily—that hadn’t quite sunk in yet.

Participant #8

A total of 8 women described learning about their *risk* of breast cancer. These women stated that they now understood the factors contributing to *risk*, such as genetics, personal history, family history, and lifestyle factors. One woman appreciated the way *RealRisks* presented *risk* information in a story format. Another woman who had a pathogenic variant in the *BRCA*2 gene felt that *RealRisks* was thought provoking. The women learned actions that they could take to reduce their risk and shared the following:

Oh, it made me think about what the risks are, specifically having to do with my situation and being in a high-risk category...Well, I guess I surmised that I was high risk, given my family history. But it solidified that, and it also taught me about options that were available to me should I choose them to reduce my risk.

Participant #15

I like the way that it was set up, as a possibility of being at risk, and that was for me, a sensitive issue...I remember there was a story—the way that it was presented by story. So it became more real rather than just the data, medical data, and to try to identify through the just numbers. It was like real.

Participant #17

#### Subtheme 2: Informed Decision Making

Most women (17/21, 81%) who used *RealRisks* reported that the DA played a role in informing their decision making about chemoprevention. The women reviewed the modules in *RealRisks* and became familiar with the options that exist to reduce their breast cancer risk, including developing an understanding of the benefits and risks of taking a chemoprevention pill. The women explained:

Yes, because the information that was provided was thorough. I mean, there was plenty of it. And I feel comfortable that—I’ve seen both sides of it. I could see why a person would take it. I believe I have made an informed decision.

Participant #6

I think it is because I did not know about it [chemoprevention] until I saw RealRisks. But definitely it played into it [my decision-making]. And I didn’t really look into it because I thought I wasn’t really the best person to be eligible for it. And now I know I do have an elevated risk.

Participant #20

Although 95% (20/21) of the women in the study viewed themselves as *healthy* and decided not to take chemoprevention, their experience with using *RealRisks* informed an autonomous decision about whether chemoprevention was the right choice for them:

I made a decision for who I am and what I want right now. So I didn’t ask my doctor. I didn’t ask anyone. I just made the decision by myself. I just took my decision because I don’t want to put anything in my body. That’s it.

Participant #9

I remember when they talked about the differing types of procedures you could go through to help prevent breast cancer. It was in-depth and I read it all but I was not in agreement with taking any kind of medicine...It was very informative, and I think if I was in poorer health, I would probably be willing to try anything. But my health is relatively good.

Participant #22

### Theme 3: Recommendations for Improvement

In addition to positive perceptions about *RealRisks,* some women (9/21, 43%) pointed out aspects of the DA that they did not like and shared recommendations for improvement.

#### Subtheme 1: Tailored for a Hispanic or Latina Population

Some women (4/9, 44%) who were not of Hispanic or Latina descent were concerned that *RealRisks* appeared to be tailored for Hispanic or Latina women. The women recognized that it was difficult to design a tool for diverse target audiences, especially those with varying health literacy and numeracy. One woman felt that *RealRisks* was designed for people who were not native English speakers. Another woman who was not of Hispanic descent was confused as to why Hispanic characters were used in the illustrations. In addition, 3 women shared that the comics were not appealing to them:

It was trying so hard to be ethnically diverse. It felt like it might have been for people where English was not their original language.

Participant #8

Found it interesting that if you’re going to draw pictures of—I actually discussed this with my doctor. If you’re going to draw pictures of people, it’s hard not to racially profile. Even though it was a line drawing, it clearly looked like a person of color. So I mean, not that it meant any good or bad to me.

Participant #24

A few women (3/9, 33%) felt that the DA made assumptions about where they were in the care continuum and did not give them the opportunity to input their own risk factors. For instance, women shared the following:

There was not an opportunity to discuss why I didn’t think I was at risk. I felt that there were assumptions made about me without even simply asking. For example, cigarette smoking causes a great deal of cancers and I do not smoke.

Participant #6

Some of it did not apply to me because I was already under care. I was already diagnosed with a BRCA2 gene mutation and already had surgery and already in the middle of this. But it wasn’t quite flexible enough...I sort of stepped into the RealRisks halfway through if you know—I didn’t need to assess my risk.

Participant #8

#### Subtheme 2: Difficult Terminology

In total, 33% (3/9) of women felt that *RealRisks* included difficult terminology, and the content was not written in layman’s terms. Of these 3 women, 2 (66%) stated that if they were not intelligent, it would have been difficult to understand the information in *RealRisks.* The participants explained the following:

I did find some of the terminology a little difficult, because I wasn’t familiar with some things. I do remember like, having to look some things up, particularly when it came to the treatment options.

Participant #7

The language used in RealRisks was not layman’s language. If I wasn’t intelligent, I wouldn’t have understood it all. Maybe it was like reading The Times paper.

Participant #22

For me, being a white, middle-class, Jewish college-educated woman, I had no trouble with it. But I wouldn’t want to speak for other cultures. I am married to a Puerto Rican man and like I could see where his mother, if she were reading this, would’ve really—she might’ve struggled...I think it tried to be very clear and very easy to understand, but I don’t know, I think it can be hard for people—sometimes the way people have a block on math, sometimes when they read about medical things they get a bit of a block.

Participant #23

#### Subtheme 3: Strong Emphasis on Chemoprevention

Overall, 22% (2/9) of women felt that the *RealRisks* DA placed a strong emphasis on chemoprevention drugs*.* Despite the importance of these drugs, these 2 women felt that they would have preferred more emphasis on the lifestyle and environmental factors affecting breast cancer risk:

I have two graduate degrees. I’m not saying this to brag. I taught college for many years. So I thought the graphics and the cartoons and all of that, were useful in general, not necessarily for myself. I felt a little bit that the chemoprevention was being pushed.

Participant #5

I felt, throughout the whole process that it was very much pushing some type of drug on me, that I felt I wasn’t really ready to consider that.

Participant #24

### Theme 4: Information Needs

Participants were asked “what additional information do you need to make decisions about reducing your breast cancer risk?” With respect to women’s information needs, 33% (7/21) of women described at least one unmet need. One woman wanted to learn more about mammography screening:

I was having this conversation with my sister recently. Because so much of the information around mammography and the frequency of mammography is changing. And so I was just having this conversation with her, that, you know, should I still be getting a yearly mammogram, I know that, age-wise, I think someone my age, and probably, with our risk, we should be getting it. But I feel like, because a lot of the information is changing, there is some confusion in terms of trying to make a decision about certain things.

Participant #5

Other women were interested in learning more about breast cancer risk factors, including modifiable lifestyle factors. Some women still desired to understand their individual breast cancer risk and what puts a woman at high risk of developing breast cancer. For example, women shared the following:

Well, I think the only information I would need or want would be during the course of my visits with my doctor is to go over my lifestyle, and just to find out if there’s any additional information that’s become available. And if there’s any additional thoughts on the benefits or risks of taking the medications. I would always consider it as I get older.

Participant #26

It’s a good question. I guess, printed materials probably, maybe like how to avoid putting myself in harm’s way, stuff that you can prevent. You know, I mean, of course there’s a lot of stuff that you can’t prevent. But I mean, if it proves that, if there was a reason for them to say, “Oh, yeah, you’re at really high risk,” I, you know, I would do whatever and apply what was suggested.

Participant #4

Probably just you know, a one-page outline of all the risks, you know, maybe just outlining everything in a concise way so that I could look at it and just know what the risks are and just have it—just be more conscious of what the risks are on a day-to-day basis and without having to go through a lot of reading and stuff.

Participant #18

## Discussion

### Principal Findings

In our racially and ethnically diverse sample of women at high risk of developing breast cancer, all participants reported that they used the web-based DA *RealRisks*. The high-risk women who participated in the intervention arm of the chemoprevention trial and were granted access to the DA reported that they generally liked the DA and considered it to be helpful. This result confirms the need for decision support for women at a high risk of developing breast cancer. In line with our objective to understand the perceptions of women at high risk of developing breast cancer regarding their experience with using *RealRisks,* our analysis generated 4 themes:

1. The acceptability of the intervention: women found the tool to be acceptable, easy to navigate, user-friendly, and easily accessible on the web.
2. Specifically endorsed elements of the DA: the DA was favored for its ability to improve knowledge and inform decision making.
3. Recommendations for improvement: participants wanted more tailoring based on user characteristics.
4. Information needs: participants reported wanting to learn more about mammography screening and breast cancer risk factors, including modifiable lifestyle factors.

Regarding theme 1, participants found *RealRisks* to be an acceptable intervention and had positive attitudes overall. The web-based nature of the intervention was very appealing to participants who appreciated being able to access the tool at their own convenience. In addition, the tool was acceptable to women in a variety of age groups, as the average age of our participants was 58.6 years (SD 10.1). Regarding theme 2, the women reported that *RealRisks* increased their knowledge about chemoprevention. This finding is aligned with the results of our pilot study, which found an increase in chemoprevention knowledge after exposure to *RealRisks* [22]. The observation that most women were autonomously motivated to make an informed decision to not take chemoprevention after using the *RealRisks* DA is aligned with the SDT, the underlining premise of the DA [35]. SDT, a theory of human motivation, emphasizes the extent to which behaviors are relatively autonomous, originating from oneself without pressure or coercion by interpersonal forces [35]. Therefore, implementing the DA in primary care may not be appropriate for all high-risk women and should be reserved for women who are undecided about taking chemoprevention and would like to further discuss the pros and cons and chemoprevention options with their primary care provider. Our results indicate that the DA offered great decision support to high-risk women, improved their knowledge, and informed their decision making about chemoprevention.

Regarding theme 3, despite the general acceptability of the DA, high-risk women wanted a more holistic approach to reducing their breast cancer risk based on tailoring that incorporates user characteristics [36]. The women felt that the DA was not personalized and made assumptions about where they were in the care continuum, which did not give them the opportunity to input their own risk factors. Other women felt that the DA placed a strong emphasis on chemoprevention drugs. Regarding theme 4, our results demonstrate that, based on the information needs reported, the high-risk women enrolled in our study wanted to learn more about general breast cancer risk factors and breast cancer screening strategies, including mammographic frequency, in addition to chemoprevention. Existing evidence suggests that interventions that are tailored based on unique characteristics and provide personalized feedback, guidance, and motivation to users might assist them in engaging in more active lifestyles [37-39] and are an effective method of promoting mammography adherence [40]. Therefore, based on our findings, future iterations of *RealRisks* may consider incorporating tailoring with a broader focus on breast cancer risk reduction and should incorporate guidance on other breast cancer risk factors.

### Strengths and Limitations

Our study has several important strengths and limitations. A strength of this study is that we had about 40% (8/21, 38%) racial and ethnic minorities, which is representative of the US population. However, we only included participants from the intervention arm of the chemoprevention trial who had accessed the tool, on average, 1 year earlier and, therefore, may have experienced recall bias. In addition, this study was conducted at a large urban academic medical center, which may not be generalizable to other geographic and practice settings.

### Future Work

Future studies should focus on developing interventions that target the knowledge gaps identified in this study and meet the needs of high-risk women to empower them to make informed decisions about reducing their breast cancer risk. Our research group is currently developing a breast cancer screening module to be added to *RealRisks*, which will provide evidence-based information on mammography screening and other methods of breast imaging.

### Conclusions

This study has demonstrated the acceptability of the *RealRisks* web-based DA among a diverse group of high-risk women, with a few caveats and recommendations for improvement. Women’s perceptions about *RealRisks* were influenced by the tool’s ability to increase their knowledge about breast cancer risk and the pros and cons of taking chemoprevention drugs; they indicated that this information facilitated their informed decision making about taking chemoprevention. We found that after using the DA, women wanted a more tailored and holistic approach to reducing breast cancer risk. The findings will inform the future development of the web-based DA. Next steps include translating the results from this study into further development of the DA and optimizing the tool’s module architecture to add a breast cancer screening module.

## Appendix

Multimedia Appendix 1 Themes, subthemes, and exemplar quotes.

## Abbreviations

DA: decision aid
RCT: randomized controlled trial
SDT: self-determination theory
