# Supplementary material for: Perceptions of Racially and Ethnically Diverse Women at High Risk of Breast Cancer Regarding the Use of a Web-Based Decision Aid for Chemoprevention: Qualitative Study Nested Within a Randomized Controlled Trial
Source: J Med Internet Res. 2021 Jun 8;23(6):e23839. doi: 10.2196/23839 (PMC8262666; doi:10.2196/23839)
Supplement: Multimedia Appendix 1 [file jmir_v23i6e23839_app1.docx]

Table. Themes, sub-themes, and quotes

| Themes | Sub-Themes | Quotes |
| --- | --- | --- |
| **Acceptability of the Intervention** | General perceptions | *I felt that it was good for me to have done it [RealRisks]. And I do feel that I got information that I would not have gotten otherwise. I mean, I felt a little bit more informed. So, I'm happy about that. [Participant #18]* |
|  | Usability of the intervention | *It was pretty simple and easy to do, and I didn't feel like there were a lot of issues with using the tool and taking the surveys at all… I didn't find it to be off putting. I didn't find it to be hard to do. I didn't find it to suck up a lot of my time. [Participant #27]* |
|  | User-friendly | *It was pretty easy to understand. I have no medical background and it gave, I thought, very clear, information about how different drugs could help. And then I could answer the questions that came next. [Participant #23]* |
|  | Easily accessible online | *It was easy because I could do it online. You know, I could like start it at work. I could finish it at home. I did not have to go back and re-answer questions. I thought it was user-friendly. [Participant #1]* |
| **Specifically endorsed elements of the decision-aid** | Improved knowledge | *I knew about the existence of the drug, tamoxifen. I know it opposes estrogen. An anti-estrogen-type drug. I did not know about other drugs. So that was new information. [Participant #24]* |
|  | Informed decision-making | *Yes, because the information that was provided was thorough. I mean, there was plenty of it. And I feel comfortable that-- I've seen both sides of it. I could see why a person would take it. I believe I have made an informed decision.* [Participant #6] |
| **Recommendations for improvement** | Tailored for a Hispanic/Latina population | *Found it interesting that if you're going to draw pictures of-- I actually discussed this with my doctor. If you're going to draw pictures of people, it's hard not to racially profile. Even though it was a line drawing, it clearly looked like a person of color. So I mean, not that it meant any good or bad to me. [Participant #24]* |
|  | Difficult terminology | *I did find some of the terminology a little difficult, because I wasn't familiar with some things. I do remember like, having to look some things up, particularly when it came to the treatment options. [Participant #7]* |
|  | Strong emphasis on chemoprevention | *I have two graduate degrees. I'm not saying this to brag. I taught college for many years. So I thought the graphics and the cartoons and all of that, were useful in general, not necessarily for myself. I felt a little bit that the chemoprevention was being pushed*. *[Participant #5]* |
| **Information needs** |  | *I was having this conversation with my sister recently. Because so much of the information around mammography and the frequency of mammography is changing. And so I was just having this conversation with her, that, you know, should I still be getting a yearly mammogram, I know that, age-wise, I think someone my age, and probably, with our risk, we should be getting it. But I feel like, because a lot of the information is changing, there is some confusion in terms of trying to make a decision about certain things. [Participant #5]* |
